# Synthesis of Phenanthrene/Pyrene Hybrid Microparticles: Useful Synthetic Mimics for Polycyclic Aromatic Hydrocarbon-Based Cosmic Dust

**DOI:** 10.1021/jacs.4c04330

**Published:** 2024-07-15

**Authors:** Emma E. Brotherton, Derek H. H. Chan, Steven P. Armes, Ronak Janani, Chris Sammon, Jessica L. Wills, Jon D. Tandy, Mark J. Burchell, Penelope J. Wozniakiewicz, Luke S. Alesbrook, Makoto Tabata

**Affiliations:** † Dainton Building, Department of Chemistry, University of Sheffield, Brook Hill, Sheffield, South Yorkshire S3 7HF, U.K.; ‡ Materials and Engineering Research Institute, Sheffield Hallam University, Sheffield, South Yorkshire S1 1WB, U.K.; § School of Physics and Astronomy, University of Kent, Canterbury, Kent CT2 7NH, U.K.; ∥ School of Chemistry and Forensic Science, University of Kent, Canterbury CT2 7NZ, U.K.; ⊥ Department of Physics, Chiba University, Chiba 2638522, Japan

## Abstract

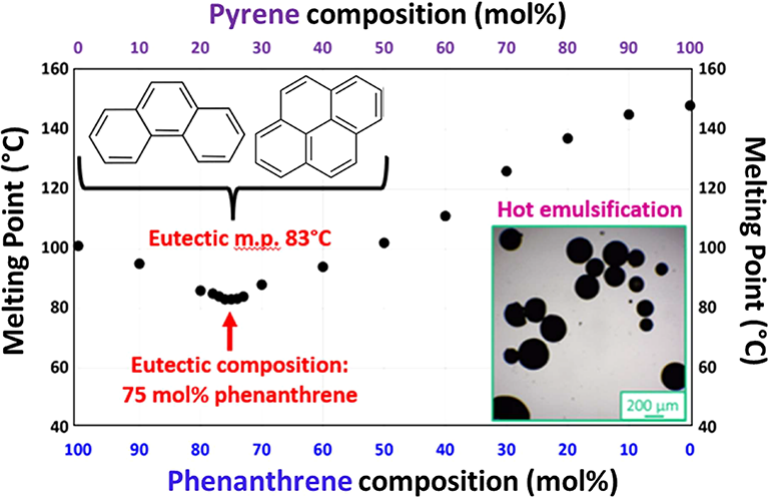

Polycyclic aromatic hydrocarbons (PAHs) are found throughout the interstellar medium and are important markers for the evolution of galaxies and both star and planet formation. They are also widely regarded as a major source of carbon, which has implications in the search for extraterrestrial life. Herein we construct a melting point phase diagram for a series of phenanthrene/pyrene binary mixtures to identify the eutectic composition (75 mol % phenanthrene) and its melting point (83 °C). The molten oil obtained on heating this eutectic composition to 90 °C in aqueous solution is homogenized in the presence of a water-soluble polymeric emulsifier. On cooling to 20 °C, polydisperse spherical phenanthrene/pyrene hybrid microparticles are obtained. Varying the stirring rate and emulsifier type enables the mean microparticle diameter to be adjusted from 11 to 279 μm. Importantly, the phenanthrene content of individual microparticles remains constant during processing, as expected for the eutectic composition. These new hybrid microparticles form impact craters and undergo partial fragmentation when fired into a metal target at 1 km s^–1^ using a light gas gun. When fired into an aerogel target at the same speed, microparticles are located at the ends of characteristic “carrot tracks”. Autofluorescence is observed in both types of experiments, which at first sight suggests minimal degradation. However, Raman microscopy analysis of the aerogel-captured microparticles indicates prominent pyrene signals but no trace of the more volatile phenanthrene component. Such differential ablation during aerogel capture is expected to inform the *in situ* analysis of PAH-rich cosmic dust in future space missions.

## Introduction

Polycyclic aromatic hydrocarbons (PAHs) are abundant throughout our Universe: they are associated with both star and planet formation, as well as providing important insights into galaxy evolution.^1−4^ In the 1980s, pioneering infrared emission observations by Tielens et al. confirmed their presence in the interstellar medium.^1^ Within our solar system, PAH molecules have been detected in meteorites^5^ and comets,^6^ on Titan,^7^ and within interplanetary cosmic dust.^8^ Furthermore, given that complex organics of varying mass^9,10^ have been detected within the plumes of water-ice grains emanating from Enceladus, it is feasible that PAH molecules may be present within the underlying ocean of Saturn’s third largest moon.

Cosmic dust typically travels at hypervelocities (>1 km s^–1^). If dust particles strike a suitable metal target at such speeds, their kinetic energy is sufficient to cause impact ionization, which produces an ionic plasma comprising atomic ions and/or molecular fragments. This enables analysis of the chemical composition of the impinging dust particles via time-of-flight mass spectrometry.^9,11−14^ This well-known principle has been used to design a series of spectrometers for various space missions, such as the CDA instrument on Cassini.^15,16^

Accordingly, laboratory-based calibration experiments for such spectrometers require synthetic mimics for cosmic dust that can attain such speeds. This can be achieved by coating the desired microscopic particles with an electrically conductive overlayer, which enables the efficient accumulation of surface charge.^17^ The resulting charged particles can then be accelerated up to the hypervelocity regime using a high-voltage van de Graaff instrument. This approach works well for many types of organic and inorganic microparticles.^18^ However, until recently, there have been no suitable synthetic mimics for PAH-rich cosmic dust.

Laboratory-based impact ionization experiments can also be performed by accelerating microparticles up to 7 km s^–1^ using a light gas gun.^19^ In such cases, no conductive coating is required and the microparticles are fired as a “buckshot”, with many near-simultaneous impact events per experiment. Such studies have been conducted for a wide range of micrometer-sized organic projectiles, which has provided valuable information with regard to impact cratering and capture processes under conditions that are comparable to those envisaged for cosmic dust collection in future space missions.^19−21^ Alternatively, icy grains of up to 50 μm have been doped with organic molecules of interest (e.g., fluorescent dyes) and fired at up to 3 km s^–1^ using a light gas gun.^22^ Similarly, 800 nm icy grains loaded with amino acids have been generated via electrospray techniques and accelerated electrostatically up to 4 km s^–1^.^23^

Recently, we reported the first synthetic mimic for PAH-based cosmic dust.^19^ Wet ball-milling of relatively coarse anthracene crystals in the presence of a suitable water-soluble polymeric dispersant produced a 20% w/w aqueous suspension of anthracene microparticles of approximately 4 μm diameter.^19^ Such microparticles were coated with an ultrathin overlayer of an electrically conductive polymer (polypyrrole) and then fired into a metal target at 1.87 km s^–1^ using a two-stage light gas gun; the resulting impact craters were examined by scanning electron microscopy.^19^ Subsequently, the same microparticles were accelerated up to 34 km s^–1^ using a van de Graaff instrument and fired at a gold target to produce characteristic time-of-flight mass spectra via impact ionization.^24^ Very recently, we utilized the relatively low melting point of phenanthrene—a structural isomer of anthracene—to prepare millimeter-sized molten droplets within a 3:1 water/ethylene glycol mixture at 106 °C. High-shear homogenization generated a relatively fine oil-in-water emulsion and subsequent cooling to ambient temperature produced polydisperse spherical phenanthrene microparticles of approximately 25 μm diameter.^20^

**Scheme 1:**
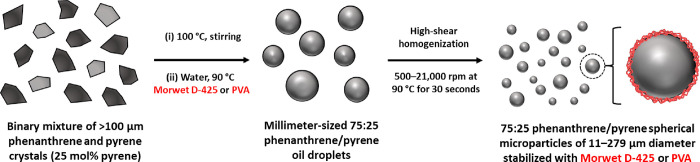
Schematic Representation of the Preparation of 75:25 Phenanthrene/Pyrene Microparticles via High-Shear Homogenization of Molten Oil Droplets in Water at 90 °C in the Presence of a Suitable Polymeric Emulsifier (Either Morwet D-425 or PVA)

**Figure 1:**
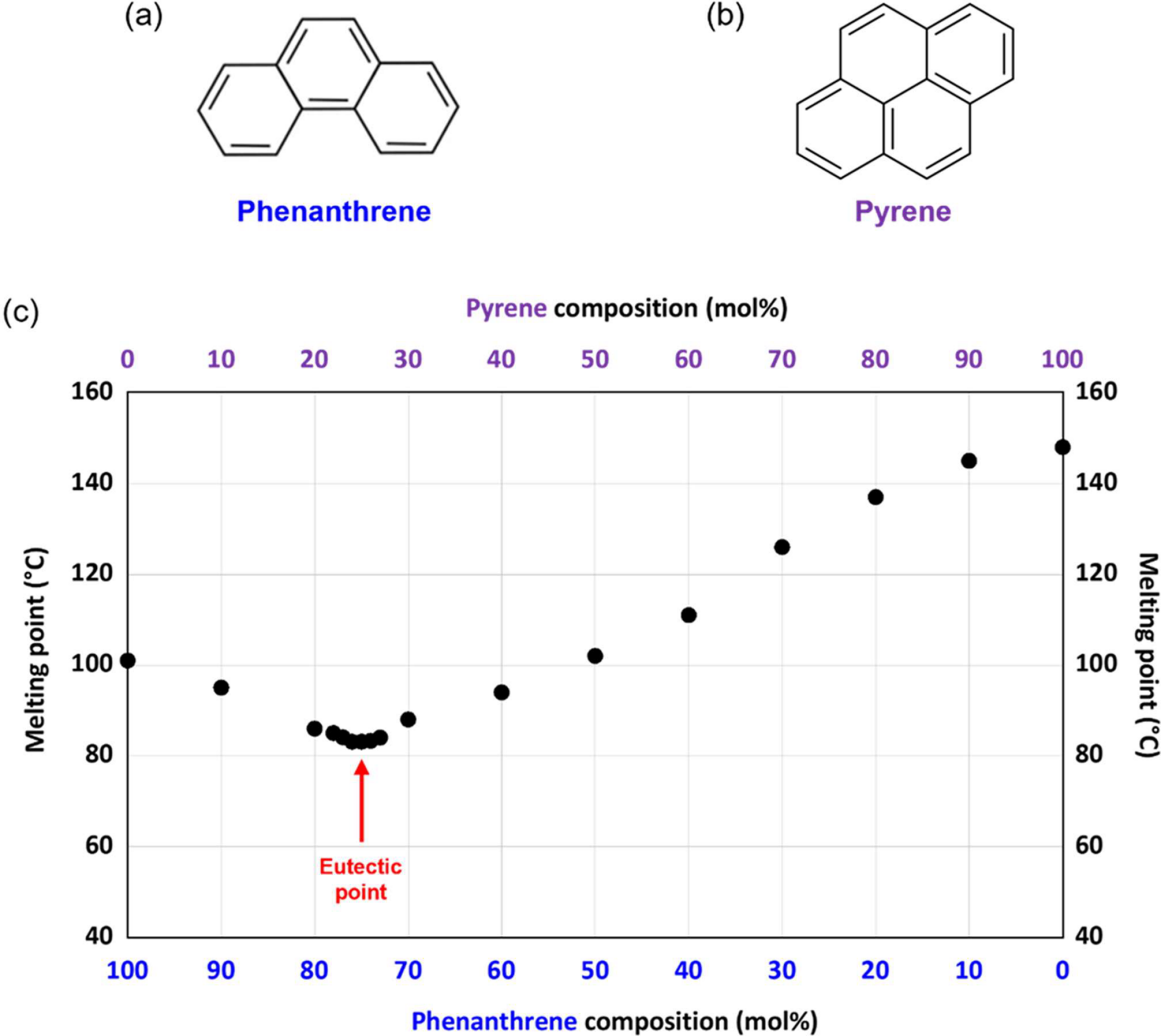
Chemical structures of the two polycyclic aromatic hydrocarbon (PAH) compounds examined in the present study: (a) phenanthrene and (b) pyrene. (c) Melting point phase diagram constructed for a series of phenanthrene/pyrene binary mixtures. The eutectic composition corresponds to 75 mol % phenanthrene, and the corresponding eutectic temperature is 83 °C.

Herein we exploit the fact that molten phenanthrene is a good solvent for pyrene, a well-known but otherwise intractable PAH (see Figure 1).^25^ This enables a series of crystalline phenanthrene/pyrene composites to be prepared simply by heating binary mixtures of these two PAHs above the melting point of phenanthrene (101 °C). Moreover, the melting points of such binary mixtures exhibit eutectic behavior (see Figure 1c), which aids their subsequent processing via hot emulsification (see Scheme 1). This is because the eutectic composition, unlike all other binary compositions, should always remain constant during such processing. Thus we envisaged that the approach developed herein should enable the rational design of model synthetic mimics for PAH-based cosmic dust grains that comprise two PAH components in the same fixed proportion for all individual microparticles. This is an important consideration for impact ionization studies conducted using a two-stage light gas gun, because it means that all such hybrid microparticles can be assumed to possess identical chemical compositions.

## Experimental Section

### Materials

Phenanthrene (98%) was purchased from Thermo Scientific (UK). Pyrene (98%) was purchased from Alfa Aesar (UK). Poly(vinyl alcohol) (PVA; nominal molecular weight = 31,000–50,000 g mol^–1^; residual vinyl acetate content = 12 mol %) and anhydrous ethylene glycol (99.8%) were purchased from Sigma-Aldrich (UK). Deionized water obtained from an Elga Medica DV25 unit was used for all experiments. Morwet D-425 was kindly provided by Syngenta (Jealotts Hill R & D site, UK).

### Synthesis

#### Preparation of Spherical 75:25 Phenanthrene/Pyrene Microparticles

Phenanthrene (0.75 g) and pyrene (0.28 g) were weighed in a 50 mL round-bottom flask. The emulsifier (Morwet D-425, 0.21 g) and deionized water (19.4 g) were added to a separate 50 mL round-bottom flask. Both flasks were sealed with rubber septa, and a metal cannula was inserted in each septum to connect the two flasks. The 75:25 phenanthrene/pyrene mixture was heated to 100 °C and stirred at 100 rpm until the crystals melted to form a homogeneous molten liquid. At this point, the temperature of the molten phenanthrene/pyrene was adjusted to 90 °C. Separately, the aqueous emulsifier solution was also heated to 90 °C and then transferred via a cannula to the round-bottom flask containing the molten phenanthrene/pyrene. For the synthesis of smaller microparticles (11 to 42 μm diameter), an IKA Ultra-Turrax T-18 homogenizer equipped with a 10 mm dispersing tool was lowered into the flask until the dispersing tool head was completely covered. Homogenization of the oil–water mixture was conducted for 30 s at a stirring rate ranging between 6000 and 21,000 rpm. For the synthesis of larger microparticles (e.g., 279 μm diameter), PVA emulsifier (0.21 g) was employed instead of Morwet D-425 using a much slower stirring rate of 500 rpm (with a magnetic flea, rather than the Ultraturrax) for 1 min.

In both cases, the resulting hot (∼90 °C) milky-white emulsion/dispersions were vacuum-filtered using a Buchner funnel, and the moist white solid was quickly redispersed in deionized water (25 mL) prior to analysis by optical microscopy and laser diffraction. Selected turbid aqueous dispersions were then freeze-dried overnight to produce fine white, free-flowing powders for further spectroscopic and hot-stage optical microscopy studies.

#### Preparation of Spherical Phenanthrene Microparticles

The synthesis of ∼200 μm phenanthrene particles was conducted using a similar protocol to that described previously.^20^ Briefly, phenanthrene (1.00 g), PVA (0.20 g), deionized water (14.1 g), and ethylene glycol (4.7 g) were added to a 50 mL round-bottom flask. A Findenser air condenser was attached to this flask, and the flask was immersed in an oil bath set at 105 °C with an initial stirring rate of 100 rpm. Under these conditions, the phenanthrene crystals melt to form molten millimeter-sized droplets. Homogenization of the resulting oil/water−ethylene glycol mixture at 105 °C was conducted by adjusting the stirring rate to 500 rpm for 1 min. The resulting hot milky-white emulsion/dispersion was vacuum-filtered using a Buchner funnel, and the moist white solid was quickly redispersed in deionized water (25 mL). The final milky-white dispersion was freeze-dried to produce a fine white powder.

### Characterization Methods

#### Laser Diffraction

Microparticles were analyzed using a Malvern Mastersizer 3000 laser diffraction instrument equipped with a Hydro EV wet dispersion unit, a red He–Ne laser (λ = 633 nm), and a blue LED light source (λ = 470 nm). The stirring rate was set at 1500 rpm, and the mean particle diameter, *D*(50), was calculated by averaging over three measurements.

#### Optical Microscopy

Representative images of the microparticles were recorded using a Cole-Palmer optical microscope fitted with a Moticam camera linked to a PC with Motic Images Plus 3.0 software.

#### Hot-Stage Optical Microscopy

Microparticle arrays were imaged using a Zeiss Axio Scope A1 microscope equipped with a Zeiss Axio Cm1 camera. A thin copper sheet (0.15 mm thickness) was placed on a Linkam THMS600 heating stage (Linkam Scientific Instruments, Tadworth, UK) attached to a T95 system controller. The particles were arranged on the copper sheet using a microspatula, and the stage was covered with three glass microscopy slides to eliminate air currents. Two LED bedside lamps (Navlinge, IKEA, UK) were used to illuminate the microparticle array at an angle of approximately 45°. A digital image of this experimental setup is shown in the Supporting Information (see Figure S1). Videos of microparticle arrays were recorded at 2.5× magnification using a heating rate of 0.5 °C min^–1^. Experiments were performed using both 75:25 phenanthrene/pyrene hybrid microparticles (mean diameter = 279 μm) and pure phenanthrene microparticles (mean diameter = 202 μm).

#### Fluorescence Microscopy

Representative images of the microparticles were recorded using a Zeiss Axio Scope A1 microscope equipped with a Zeiss Axio Cm1 camera. Fluorescence microscopy images were obtained using a LED radiation source combined with filter set 02 (excitation λ = 365 nm; emission λ > 420 nm). Images were taken at 2.5× magnification.

#### ^1^H NMR Spectroscopy

Spectra were recorded for 279 μm diameter 75:25 phenanthrene/pyrene particles, pure phenanthrene, and pure pyrene in *d*_6_-acetone using a 400 MHz Bruker Avance-400 spectrometer at 298 K with 16 scans being averaged per spectrum. The integrated proton signals at 7.60–7.75 ppm assigned to four C–H groups for phenanthrene were compared to proton signals at 8.20 ppm corresponding to four C–H groups for pyrene.

#### Raman Microscopy Studies of 279 μm-Diameter 75:25 Phenanthrene/Pyrene Microparticles

Spectra were acquired on a Renishaw inVia Raman microscope fitted with a 1200 lines/mm grating and a Peltier-cooled CCD array detector (1024 × 256 pixels) using a laser excitation wavelength of 785 nm. The laser power was varied between 0.2 and 2.0 mW, which corresponds to approximately 0.1–1.0% of the maximum laser power of 198 mW. Exposure times were varied between 1 and 10 s, with shorter times corresponding to the lowest laser power. These conditions were chosen to minimize the fluorescence background and sample degradation, respectively. All samples were placed on a silicon wafer substrate prior to imaging and spectroscopic analysis.

A 5× objective lens was selected, which corresponds to a slot size of approximately 9.6 μm. Typically, 10–20 partial spectra were co-added for the individual 75:25 phenanthrene/pyrene microparticles (mean diameter = 279 μm), while high-quality representative spectra for the phenanthrene, pyrene, and 75:25 phenanthrene/pyrene bulk powder reference materials were obtained within 1–2 scans.

For quantification purposes, the 709 cm^–1^ band assigned to a torsional mode of the C11–C12–C13 subunit for phenanthrene^26^ was compared to the 591 cm^–1^ band corresponding to an in-plane C–H bending mode for pyrene.^27^ Peak areas for these two bands were calculated via peak fitting using WiRE version 3.4 software supplied by the instrument manufacturer. The same software was used for baseline correction to eliminate the underlying fluorescence background.

#### Line Scan Experiment Conducted on a Single Microparticle

Using a 5× objective lens, spectra were recorded at eleven equally spaced points at 30 μm intervals across a single 75:25 phenanthrene/pyrene microparticle of approximately 360 μm diameter. Ten spectra were averaged at each point, and the relative areas for the 709 and 591 cm^–1^ bands were determined by peak integration in each case.

#### Light Gas Gun Experiments Using 75:25 Phenanthrene/Pyrene Microparticles

Hypervelocity experiments were conducted using a two-stage light gas gun at the University of Kent, UK.^28,29^ This gun fires a sabot, which is discarded in flight. The sabot was loaded before each shot with the desired 75:25 phenanthrene/pyrene hybrid microparticles (approximately 1 mg; 279 μm diameter). These projectiles were fired at the target, which was placed in a vacuum chamber held at 0.6 mbar for each shot. The shot speed was determined using timing signals from the sabot as it emerges from the gun barrel and is intercepted near the target; this approach provides an accuracy of ±4%. Two types of experiments were performed (see Scheme S1 in the Supporting Information for a schematic illustration in each case): (i) using a metal target of aluminum foil (type Al-1080, 100 μm thickness, placed on an aluminum mounting stub) at 0.96 km s^–1^; (ii) using a silica aerogel target at 0.99 km s^–1^. The latter target was rectangular in shape, with an areal face of 18.3 mm × 33.2 mm, a length of 72.0 mm, and a density of 91.5 kg m^–3^. This highly porous, low-density transparent aerogel means that the impinging microparticles experience a relatively low shock pressure upon impact and can be captured intact at the end of “carrot tracks” for subsequent *in situ* analysis.^30^ Such targets have been previously used as a capture medium for cosmic dust.^31−33^

Post-shot optical microscopy studies were performed using a Leica MZ16F microscope equipped with a Leica K3C camera. Bright-field b/w images were recorded using a Leica CLS 150× light source to illuminate the sample. Fluorescence images were recorded using the fluorescence mode of the Leica MZ16F microscope and using a Fisher Scientific UVP Blak-Ray B-100AP/R high-intensity UV lamp (λ = 365 nm) to illuminate the aerogel.

#### Raman Microscopy Studies of 279 μm-Diameter 75:25 Phenanthrene/Pyrene Microparticles Captured within an Aerogel Target

Spectra were acquired using a LabRam HR Raman microscope fitted with a 600 lines/mm grating and a Peltier-cooled CCD array detector (1024 pixels × 256 pixels). The laser excitation wavelength was 633 nm, and the laser power was varied between 5 and 12.5 mW. A 10× objective was employed, and the corresponding spot size was typically 3 μm, which is much less than the mean particle diameter. The spectral accumulation time was varied from 5 to 12 s, with 10–35 accumulations per spectrum.

## Results and Discussion

### Synthesis of 75:25 Phenanthrene/Pyrene Microparticles of 11 to 42 μm Diameter

A melting point phase diagram constructed for a series of phenanthrene/pyrene binary mixtures is shown in Figure 1. These data are in good agreement with the literature.^25^ Notably, the melting point of a 75:25 phenanthrene/pyrene binary mixture is 83 °C, which is significantly lower than that of phenanthrene alone (101 °C). Moreover, this eutectic temperature is well below the normal boiling point of water (100 °C). In principle, this enables convenient emulsification of this eutectic composition via high-shear homogenization in purely aqueous solution, i.e., without requiring the addition of high boiling point co-solvents such as ethylene glycol.^20^

**Figure 2:**
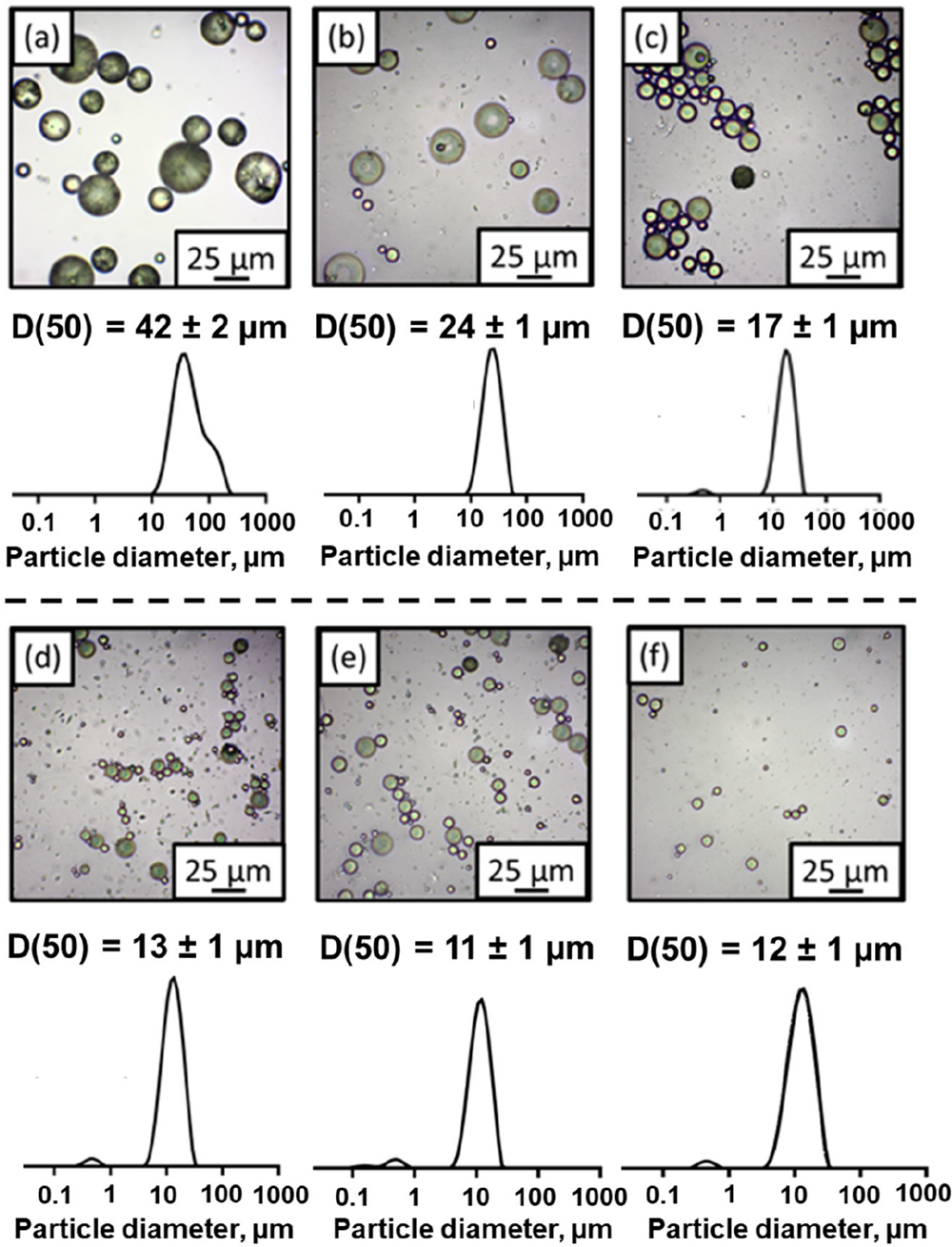
Laser diffraction data and optical microscopy images obtained for 75:25 phenanthrene/pyrene microparticles prepared via hot emulsification at a stirring rate of (a) 6000 rpm, (b) 9000 rpm, (c) 12,000 rpm, (d) 15,000 rpm, (e) 18,000 rpm, and (f) 21,000 rpm.

**Figure 3:**
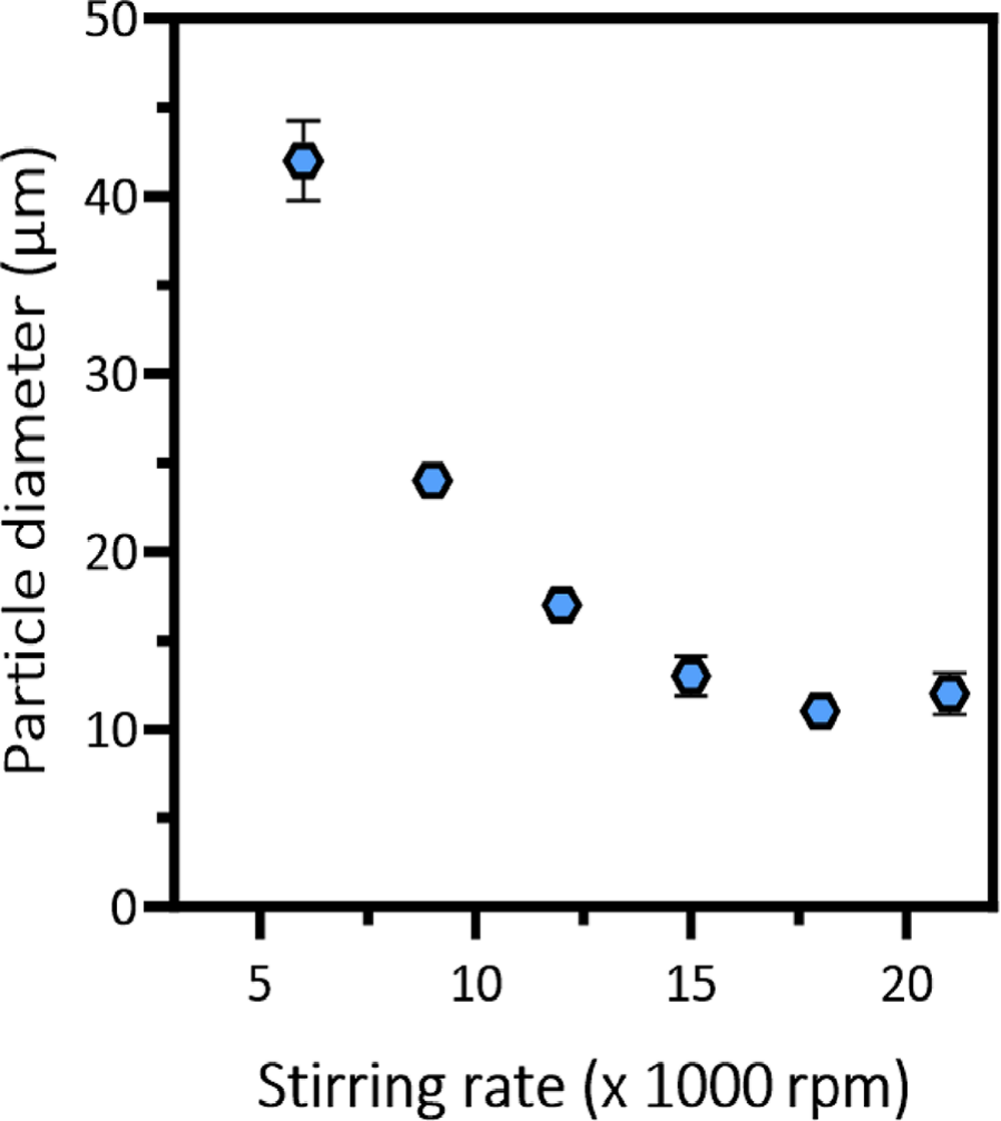
Effect of varying the stirring rate on the mean particle diameter (*D*_50_) reported by laser diffraction for a series of 75:25 phenanthrene/pyrene microparticles prepared at 90 °C via the hot emulsification route shown in Scheme 1 using the Morwet D-425 emulsifier.

Indeed, heating this 75:25 phenanthrene/pyrene eutectic composition in aqueous solution up to 90 °C produces millimeter-sized molten phenanthrene/pyrene droplets. High-shear homogenization of this hot mixture affords microscopic droplets that can be stabilized using an anionic commercial water-soluble polymer (Morwet D-425)^19^ as an emulsifier (see Scheme 1). Cooling this hot oil-in-water emulsion produces spherical 75:25 phenanthrene/pyrene microparticles. Systematic variation of the stirring rate enables the mean diameter of such microparticles to be varied from approximately 11 to 42 μm diameter, as determined by optical microscopy and laser diffraction studies (Figure 2). The inverse relationship between the stirring rate and the microparticle diameter is plotted in Figure 3. As expected, smaller microparticles are obtained when using faster stirring rates. For example, a stirring rate of 18,000 rpm affords 75:25 phenanthrene/pyrene microparticles of 11 μm diameter, whereas a stirring rate of 6000 rpm affords 75:25 phenanthrene/pyrene microparticles of 42 μm diameter. In principle, systematic variation of the emulsifier concentration should also provide control over the mean droplet diameter, but this alternative approach was not investigated in the present study.

### Synthesis of Relatively Large 75:25 Phenanthrene/Pyrene and Pure Phenanthrene Microparticles

**Figure 4:**
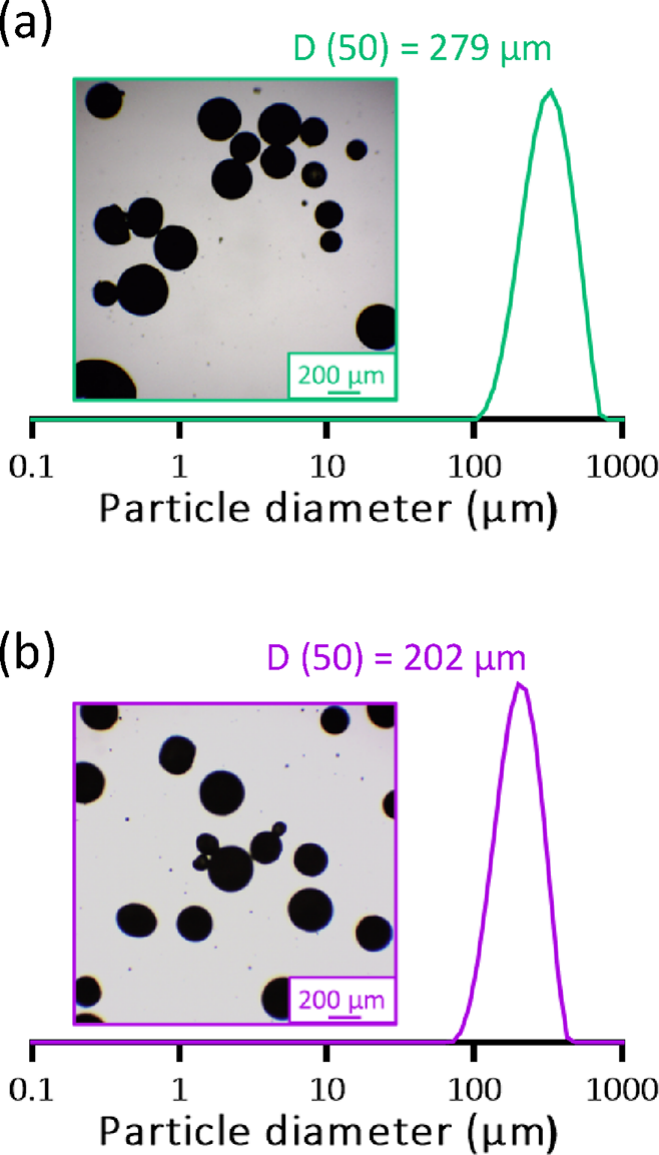
Laser diffraction particle size distribution and corresponding representative optical microscopy image obtained for (a) 75:25 phenanthrene/pyrene microparticles of 279 μm diameter prepared in water at 90 °C and (b) pure phenanthrene microparticles of 202 μm diameter prepared in a 3:1 ethylene glycol/water mixture at 105 °C. In both cases, the emulsifier was poly(vinyl alcohol), and the stirring rate was 500 rpm.

Relatively large 75:25 phenanthrene/pyrene microparticles can be prepared using an approach similar to that outlined in Scheme 1. However, in this case, an alternative emulsifier was required to produce well-defined microparticles with a unimodal particle size distribution. Accordingly, a commercial non-ionic water-soluble polymer, PVA, was employed instead of Morwet D-425 and a relatively slow stirring rate of 500 rpm was used to produce spherical microparticles of around 279 μm diameter in deionized water at 90 °C (Figure 4a). The corresponding pure phenanthrene microparticles of approximately 202 μm diameter were prepared at 105 °C in a 3:1 water/ethylene glycol mixture employing the same emulsifier and stirring rate for use as a reference material (Figure 4b). The Morwet D-425 emulsifier is a water-soluble aromatic polymer comprising anionic sulfonate groups and polymerized naphthalene units.^19^ The latter interact with the phenanthrene and pyrene molecules within the molten oil droplets via π–π* interactions, leading to strong adsorption. In contrast, the aliphatic PVA emulsifier is only weakly adsorbed at the oil/water interface, which leads to larger oil droplets under comparable conditions. Notably, such relatively large microparticles are comparable in size to the largest mass fraction of cosmic dust that falls into the Earth’s atmosphere each year.^34^

### Chemical Composition of 75:25 Phenanthrene/Pyrene Microparticles of 279 μm Diameter

**Figure 5:**
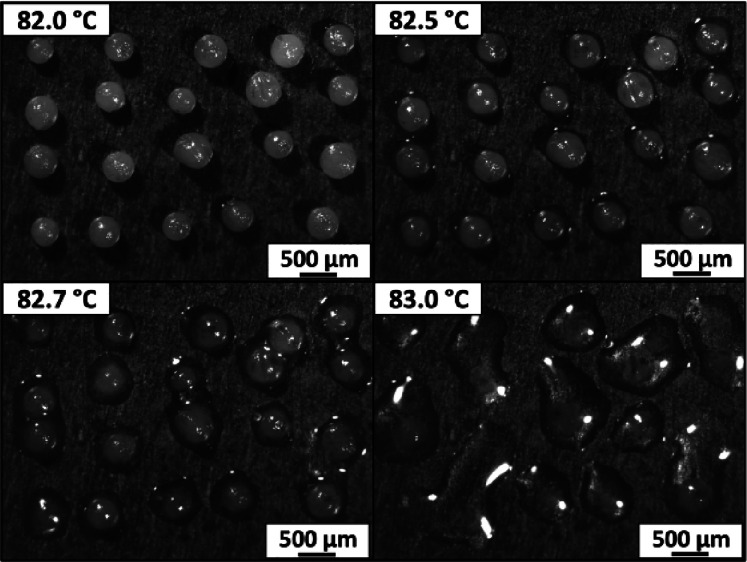
Representative video stills recorded during hot-stage optical microscopy analysis of an array comprising twenty 279 μm 75:25 phenanthrene/pyrene microparticles at 82–83 °C. The heating rate used in this experiment was 0.5 °C min^–1^. Video still images recorded for a control experiment using 202 μm phenanthrene microparticles are shown in Figure S2.

**Figure 6:**
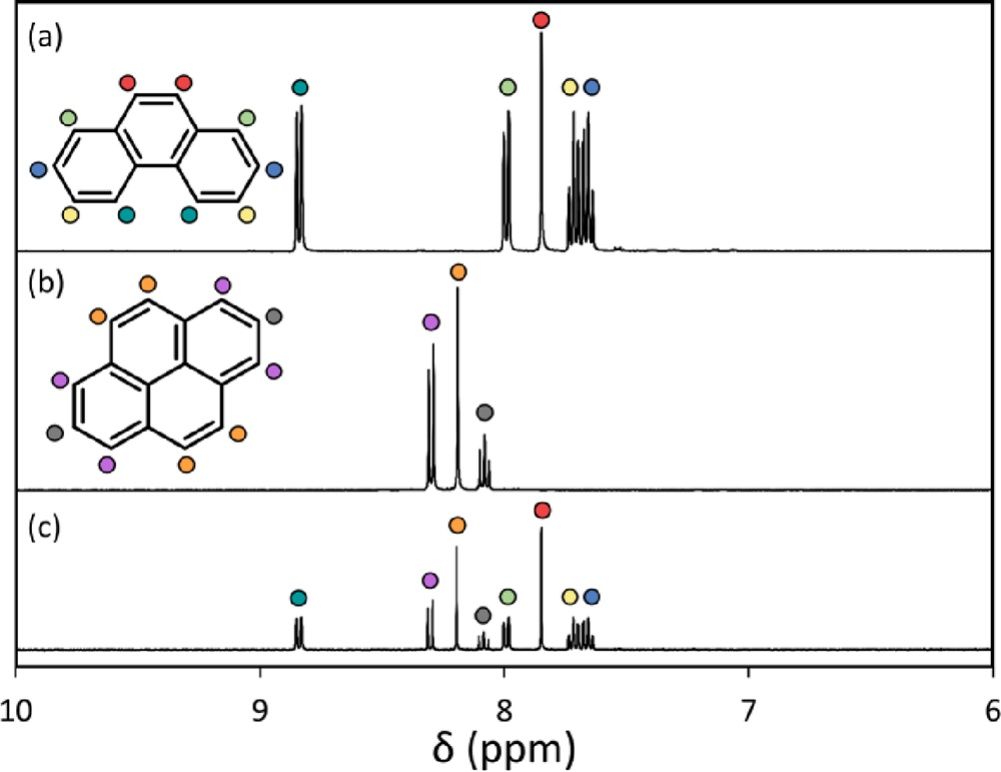
Partial ^1^H NMR spectra (*d*_6_-acetone, 400 MHz) recorded for (a) phenanthrene, (b) pyrene, and (c) an individual 75:25 phenanthrene/pyrene microparticle (mean diameter = 279 μm).

For their intended use as a synthetic mimic for PAH-based cosmic dust, it is important to demonstrate that each hybrid microparticle has precisely the same chemical composition (i.e., 75 mol % phenanthrene). In principle, the extent of any compositional heterogeneity can be assessed by observing the melting behavior of individual microparticles. Accordingly, hot-stage optical microscopy was used to record videos of arrays of twenty 75:25 phenanthrene/pyrene microparticles (mean diameter = 279 μm) placed on a copper sheet mounted on a Linkam temperature control unit (see Figure S1). Representative video stills were recorded at a constant heating rate of 0.5 °C min^–1^. An initial control experiment was performed using pure phenanthrene microparticles (mean diameter = 202 μm) to confirm that the experimental setup did not produce any unwanted thermal gradients across the copper sheet. As expected, each of these microparticles melted at the known mp for phenanthrene (101 °C; see Figure S2). Similarly, all 75:25 phenanthrene/pyrene microparticles melted at the anticipated temperature of 83 °C (Figure 5). This indicates a high degree of chemical homogeneity for these microparticles, as expected for this eutectic composition. Nevertheless, further spectroscopic evidence was sought to confirm the constant 75 mol % phenanthrene composition for such microparticles. In the case of the 279 μm diameter phenanthrene/pyrene microparticles, the mass of an individual microparticle is estimated to be approximately 0.1 mg, which should be sufficient to allow a good-quality NMR spectrum to be recorded. Accordingly, individual microparticles were dissolved in *d*_6_-acetone, which is a good solvent for both phenanthrene and pyrene, and ^1^H NMR spectra were recorded to determine their mean composition (Figure 6). ^1^H NMR spectra obtained for ten individual phenanthrene/pyrene microparticles are shown in Figure S3, and a typical example is shown in Figure 6, along with reference spectra recorded for pure phenanthrene and pure pyrene dissolved in *d*_6_-acetone. The phenanthrene/pyrene molar ratio was calculated in each case by comparing the blue and yellow signals shown in Figure 6c assigned to four phenanthrene protons (Figure 6a) to the orange signals that correspond to four pyrene protons (Figure 6b). If this microparticle contained 75 mol % phenanthrene, then the integrated signal ratio should be 3.0. Averaging over ten microparticles, the experimental ratio was calculated to be 2.9 ± 0.1. Thus the expected 75 mol % phenanthrene content of these microparticles is confirmed.

**Figure 7:**
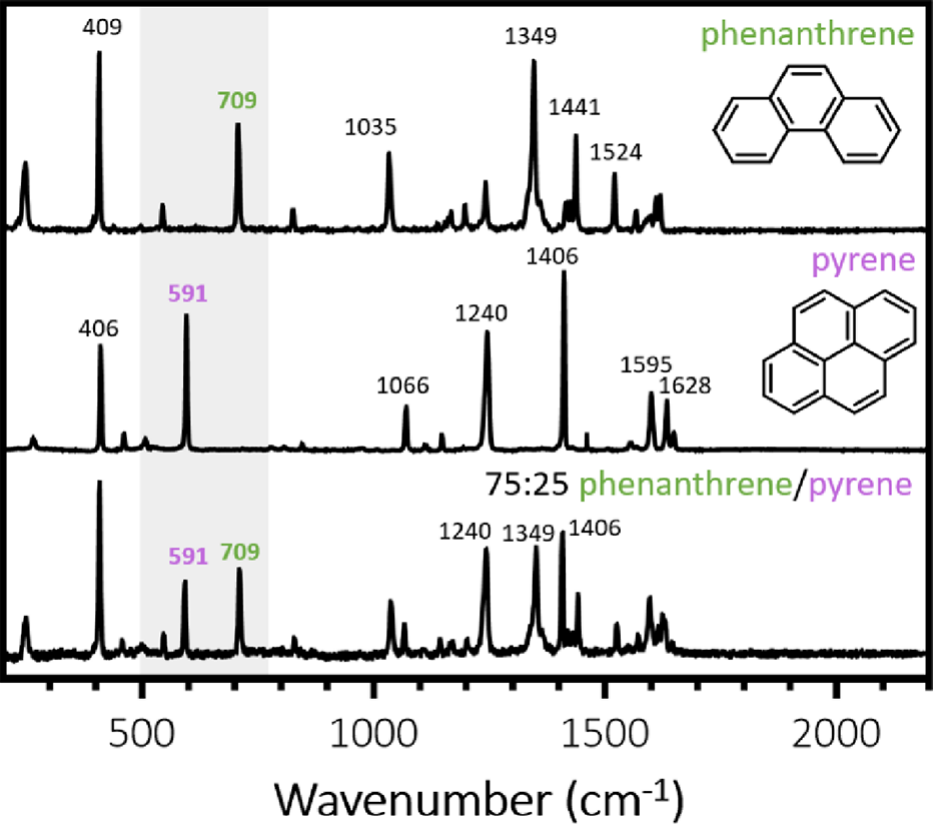
Raman spectra recorded for pure phenanthrene, pure pyrene, and a 75:25 phenanthrene/pyrene hybrid crystal obtained via melt processing (but without hot emulsification). To determine the phenanthrene content (mol %) of individual 75:25 phenanthrene/pyrene microparticles, the integrated intensity of the Raman band at 709 cm^–1^ assigned to phenanthrene was compared to that of pyrene at 591 cm^–1^ (see the main text for spectral assignments).

In principle, Raman microscopy should be suitable for determining the chemical composition of the 75:25 phenanthrene/pyrene microparticles prepared in this study. Provided that a suitable laser wavelength is selected to minimize background fluorescence,^35^ high-quality spectra can be recorded for individual microparticles, as well as for an appropriate reference material. Moreover, this analytical technique has excellent spatial resolution, which enables compositional mapping experiments to be performed on relatively large microparticles.^36,37^ The Raman spectra for phenanthrene and pyrene have been reported in the literature.^27,35^ Very similar spectra were recorded for as-supplied phenanthrene and pyrene coarse crystals, as well as for macroscopic 75:25 phenanthrene/pyrene hybrid crystals obtained via the melt processing route prior to hot emulsification (see Figure 7).

Minor differences in relative band intensities were attributed to the choice of laser excitation wavelength (λ = 785 nm).^35^ In the present study, we focused on the 709 cm^–1^ band assigned to a torsional mode of the C11–C12–C13 sub-unit for phenanthrene and the 591 cm^–1^ band assigned to the in-plane C–H bending mode for pyrene.^26,27^ These two bands were selected because they were well resolved, relatively intense, and reasonably close to each other, which meant that only partial spectra needed to be recorded. For the macroscopic 75:25 phenanthrene/pyrene hybrid crystal, the mean peak area ratio, *P*_709/591_, for these two Raman bands was calculated to be 1.34 ± 0.21 (averaged over three spectra recorded at different points).

**Figure 8:**
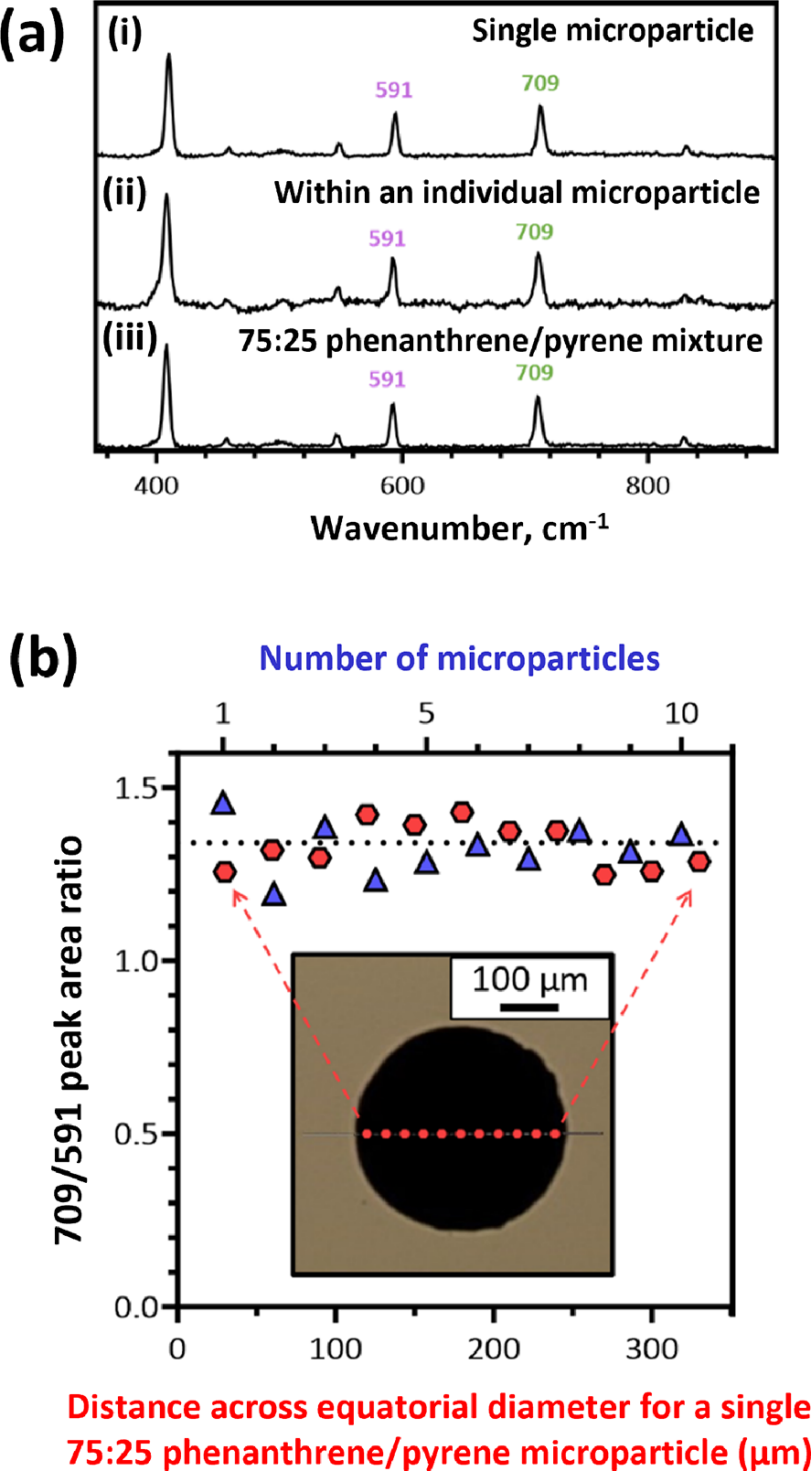
(a) Representative partial Raman spectra recorded: (i) for one of ten individual 75:25 phenanthrene/pyrene microparticles (upper spectrum); (ii) at eleven equally spaced points (steps) across the equatorial diameter of an individual microparticle (middle spectrum); (iii) for a macroscopic 75:25 phenanthrene/pyrene hybrid crystal reference material (lower spectrum). (b) Peak area ratios calculated from the 709 and 591 cm^–1^ Raman bands assigned to phenanthrene and pyrene, respectively, for (i) ten individual 75:25 phenanthrene/pyrene microparticles with a mean diameter of 279 μm (upper *x* axis, blue triangles) and (ii) eleven points along the equatorial diameter of a 360 μm 75:25 phenanthrene/pyrene microparticle (lower *x* axis, red hexagons). The inset optical image shows the equatorial diameter along which eleven Raman spectra were acquired at equally spaced points. The horizontal black dotted line indicates a peak area ratio, *P*_709/591_, of 1.34, as determined for the macroscopic 75:25 phenanthrene/pyrene hybrid crystal reference material.

The same Raman microscopy instrument was then used to record representative partial Raman spectra (from 360 to 880 cm^–1^; see Figure 8a for a representative spectrum) for a series of ten individual 75:25 phenanthrene/pyrene microparticles (mean diameter = 279 μm), and the peak area ratio, *P*_709/591_, was determined for each microparticle (Figure 8b).

A mean *P*_709/591_ of 1.33 ± 0.06 was calculated for these ten microparticles (n.b. unlike the NMR studies, *P*_709/591_ is not equal to 3.0 because this parameter depends on the scattering cross-sections for the two Raman bands as well as the phenanthrene/pyrene molar ratio). In combination with the NMR spectroscopy data, these Raman studies show that each of these microparticles possesses essentially the same phenanthrene content and hence indicate that no significant compositional drift occurs during processing via the hot emulsification route. Furthermore, eleven Raman spectra (see Figure 8a for a representative spectrum) were also acquired at eleven equally spaced points across the equatorial diameter of a single 75:25 phenanthrene/pyrene microparticle of approximately 360 μm diameter (see Figure 8b, inset). Again, *P*_709/591_ was calculated for each point, and the mean value was determined to be 1.33 ± 0.05, which is very similar to that determined for the ten individual microparticles (see above). Hence these measurements indicate a relatively uniform chemical composition across this individual microparticle.

### Fluorescence Microscopy Studies of 75:25 Phenanthrene/Pyrene Microparticles

**Figure 9:**
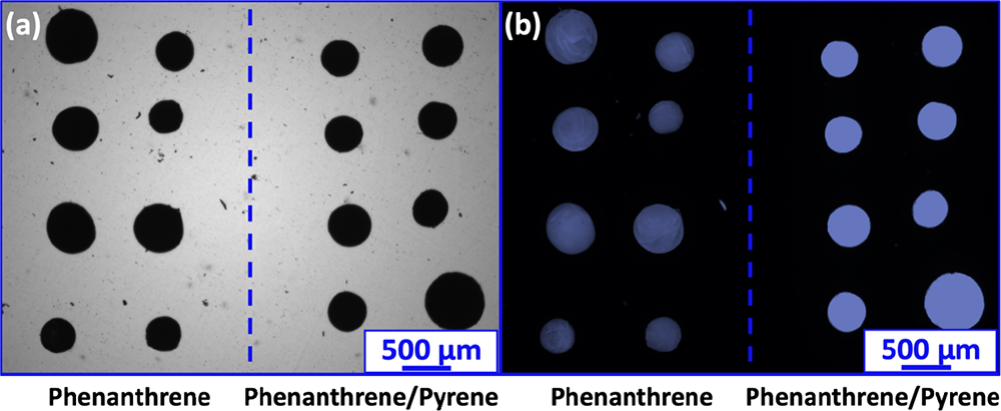
(a) Optical microscopy images and (b) corresponding fluorescence microscopy images (filter set, 02; excitation λ = 365 nm; emission λ > 420 nm) recorded for two arrays comprising eight 202 μm phenanthrene microparticles and eight 279 μm 75:25 phenanthrene/pyrene microparticles.

Recently, we reported the synthesis of phenanthrene microparticles via hot emulsification in a 3:1 water/ethylene glycol mixture at 106 °C using an alternative water-soluble polymeric emulsifier.^20^ The intrinsic autofluorescence exhibited by phenanthrene^38^ proved to be useful for the identification of phenanthrene residues associated with the impact craters that are formed when such microparticles are fired at a metal target in light gas gun experiments. This was achieved using fluorescence microscopy in reflectance mode (excitation wavelength = 365 nm; emission wavelength >420 nm). In the present study, we examined how the introduction of 25 mol % pyrene affected this autofluorescence. Accordingly, a fluorescence microscopy image was recorded for an array of eight 75:25 phenanthrene/pyrene microparticles placed next to an array of eight phenanthrene microparticles (Figure 9).

Clearly, the former microparticles are significantly brighter than the latter, which indicates that introducing 25 mol % pyrene leads to enhanced autofluorescence. The underlying physical explanation for this unexpected observation is not yet understood. Nevertheless, these new 75:25 phenanthrene/pyrene microparticles should be well suited for impact crater analyses.

### Light Gas Gun Experiments Using 75:25 Phenanthrene/Pyrene Microparticles

Two types of targets were examined in this study: aluminum foil and an ultralow-density silica aerogel. Aluminum is relatively cheap, strong, and lightweight. As such, it is widely used to construct spacecraft and is well established as a useful model target for hypervelocity impact experiments.^39^ Ultralow density aerogel targets have been used in previous space missions to capture fast-moving cosmic dust particles emanating from cometary tails intact with minimal thermal ablation. Such targets are then returned to Earth for extensive spectroscopic studies and other chemical analyses.^30−32^ Indeed, both aluminum foils and aerogel targets were successfully used for the NASA Stardust mission to collect dust particles from comet P/Wild-2. Such targets were then returned to Earth for extensive spectroscopic studies and other chemical analyses.^30−32,40^

**Figure 10:**
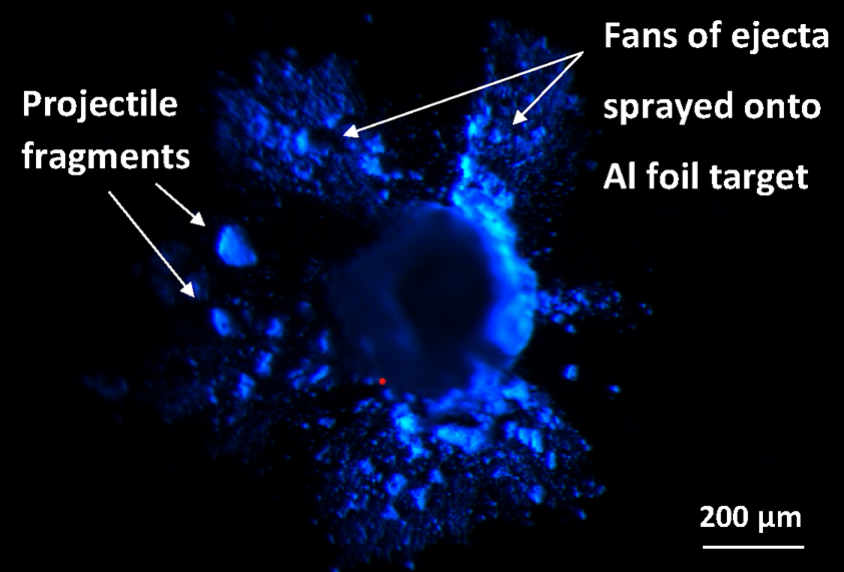
Optical image recorded for an impact crater on aluminum foil viewed under UV irradiation (λ = 365 nm). Most of the microparticle is embedded within the foil, with several corresponding fragments located on the surrounding surface (see white arrows on the left of the microparticle). Fan-like jets are visible on the target surface (see arrows above the microparticle) and are assigned as very fine ejecta fragments/molten droplets emanating from the microparticle.

After the 279 μm diameter 75:25 phenanthrene/pyrene microparticles were fired into the aluminum foil, optical microscopy was used to inspect this target for impact features. The microparticles impacted and partially penetrated the surface in various places and underwent a degree of fragmentation (see Figure 10). Excitation with UV radiation led to fluorescence emission: not only do the microparticles (plus large fragments) become visible but also fan-shaped sprays of ejecta can be observed emanating from the main impact site.

Projectile impacts on aluminum foils are believed to involve—with increasing impact velocity—the following sequence of events: rebound, rebound and fracture, sticking, sticking with fracture, cratering with projectile fragmentation, and cratering with melted residue.^21,41,42^ Depending on the projectile composition, this sequence spans a velocity regime of 0.2–3.0 km s^–1^ (which corresponds to a peak pressure of typically 0.1–10 GPa).^21^ The boundaries between this series of regimes depend not only on the impact velocity but also on projectile/target properties such as tensile strength, melting point, etc. For example, it has been reported that 4–10 μm diameter poly(methyl methacrylate) microparticles either adhere intact (stick) or rebound from aluminum foil when fired at 0.8–1.0 km s^–1^ but form craters with molten residues at 2 km s^–1^.^43^ Such prior studies serve to indicate the likely regimes for rebound and sticking (low speed) and cratering with melted residue (at the higher speed). The new data reported herein lie in the intermediate regime that involves both adhesion and partial fracturing of the projectile.

In principle, the peak shock pressure generated during such high-velocity impacts can be calculated using the planar impact approximation.^44^ This assumes that the relationship for the linear shock wave speed is of the form *U* = *C* + *Su*, where *U* is the shock speed and *u* is the projectile speed. Here *C* and *S* are coefficients that can be calculated by fits to data for a given type of material. We assume the following values: aluminum 1080 (density, 2712 kg m^–3^; *C* = 5376 m s^–1^; *S* = 1.339),^45^ phenanthrene (density, 1212 kg m^–3^; *C* = 3139 m s^–1^; *S* = 0.309),^20^ and pyrene (density, 1275 kg m^–3^; *C* = 3065 m s^–1^; *S* = 1.444).^46^ There are no comparable literature data for 75:25 phenanthrene/pyrene hybrid microparticles, so we calculate the peak pressures for phenanthrene and pyrene separately and use these results to infer the likely range for the actual peak pressure. Accordingly, for an impact on aluminum foil at 0.96 km s^–1^, the estimated peak pressures are 3.12 GPa (for phenanthrene) and 3.75 GPa (for pyrene). This range lies just above the threshold suggested for the boundary between adhesion with partial fragmentation and cratering with concomitant melting. In practice, the tensile strength and melting point of phenanthrene and pyrene are also likely to be important parameters. Nevertheless, this accounts for the observation of melt jetting away from the contact point, which is the most heavily shocked (and heated) region within an impact zone.

### Raman Microscopy Studies of Aerogel-Captured Microparticles

**Figure 11:**
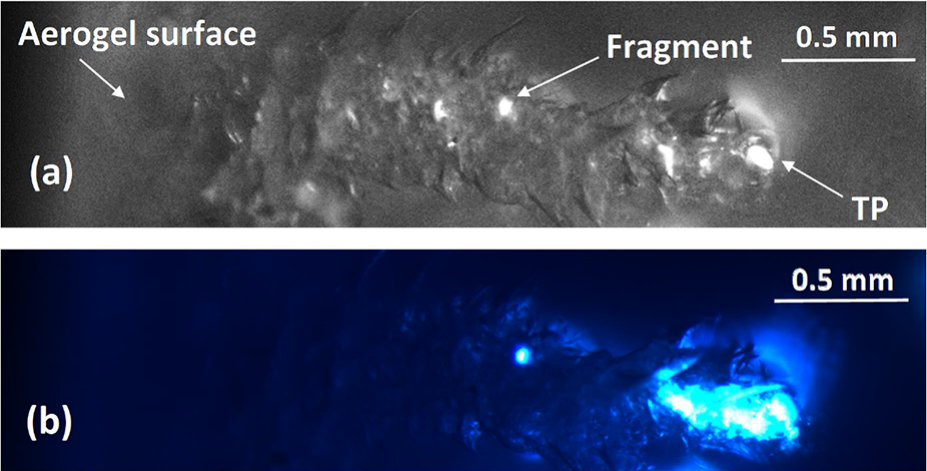
Optical image of a characteristic “carrot track” within the aerogel. (a) Normal illumination from above. The microparticle (labeled TP for “terminal particle”) entered the aerogel from the left before coming to rest at the end of the track on the right. Some fragments of the original microparticle are deposited along the track (e.g., see label). (b) UV side illumination (λ = 365 nm) of the same view of the aerogel track. Both the terminal particle and its associated large fragment exhibit strong fluorescence when viewed under such conditions.

**Figure 12:**
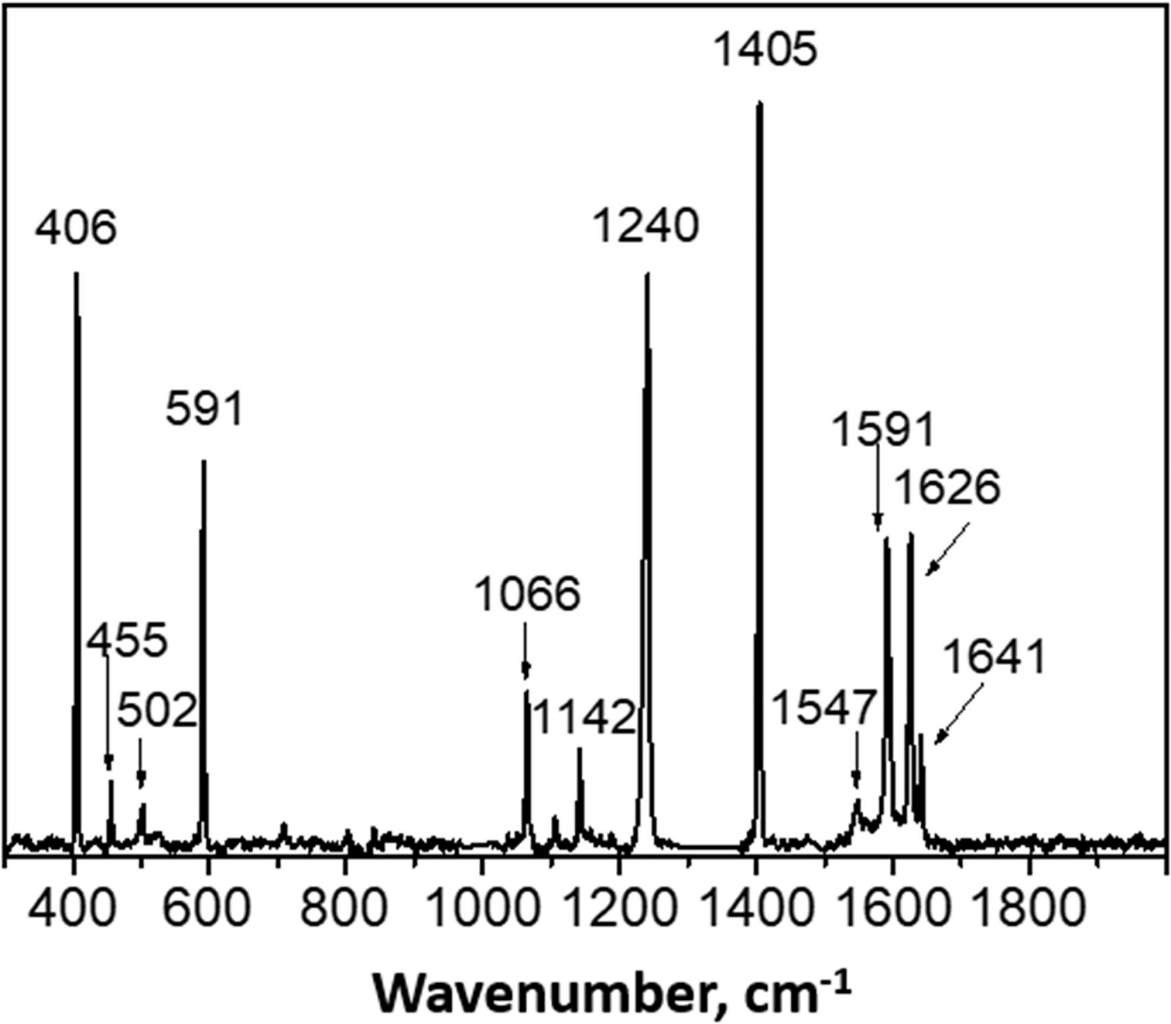
Background-subtracted partial Raman spectrum for a captured 75:25 phenanthrene/pyrene microparticle after being fired into an aerogel target at approximately 1 km s^–1^. The most prominent bands are identical (typically within ±1 cm^–1^) to those observed for pyrene in Figure 7. Minor bands can also be assigned to this pyrene spectrum. Notably, there is no indication of any phenanthrene bands.

Post-shot imaging of the aerogel target revealed a series of “carrot tracks” that are characteristic of the penetration of fast-moving microparticles into an aerogel target. One example of such a track is shown in Figure 11a. When viewed under UV irradiation (Figure 11b), the captured microparticles exhibited autofluorescence, as observed for the original particles (Figure 9). Since the aerogel target is optically transparent, this enables *in situ* analysis of the captured microparticles by Raman microscopy. When interrogated using a Raman microscope equipped with a red laser, the original microparticles gave spectra very similar to those shown in Figure 7, albeit with a strong fluorescence background owing to the shorter laser wavelength. Microparticles captured within the aerogel were then analyzed and a typical partial Raman spectrum (300–2000 cm^–1^) is shown in Figure 12.

**Figure 13:**
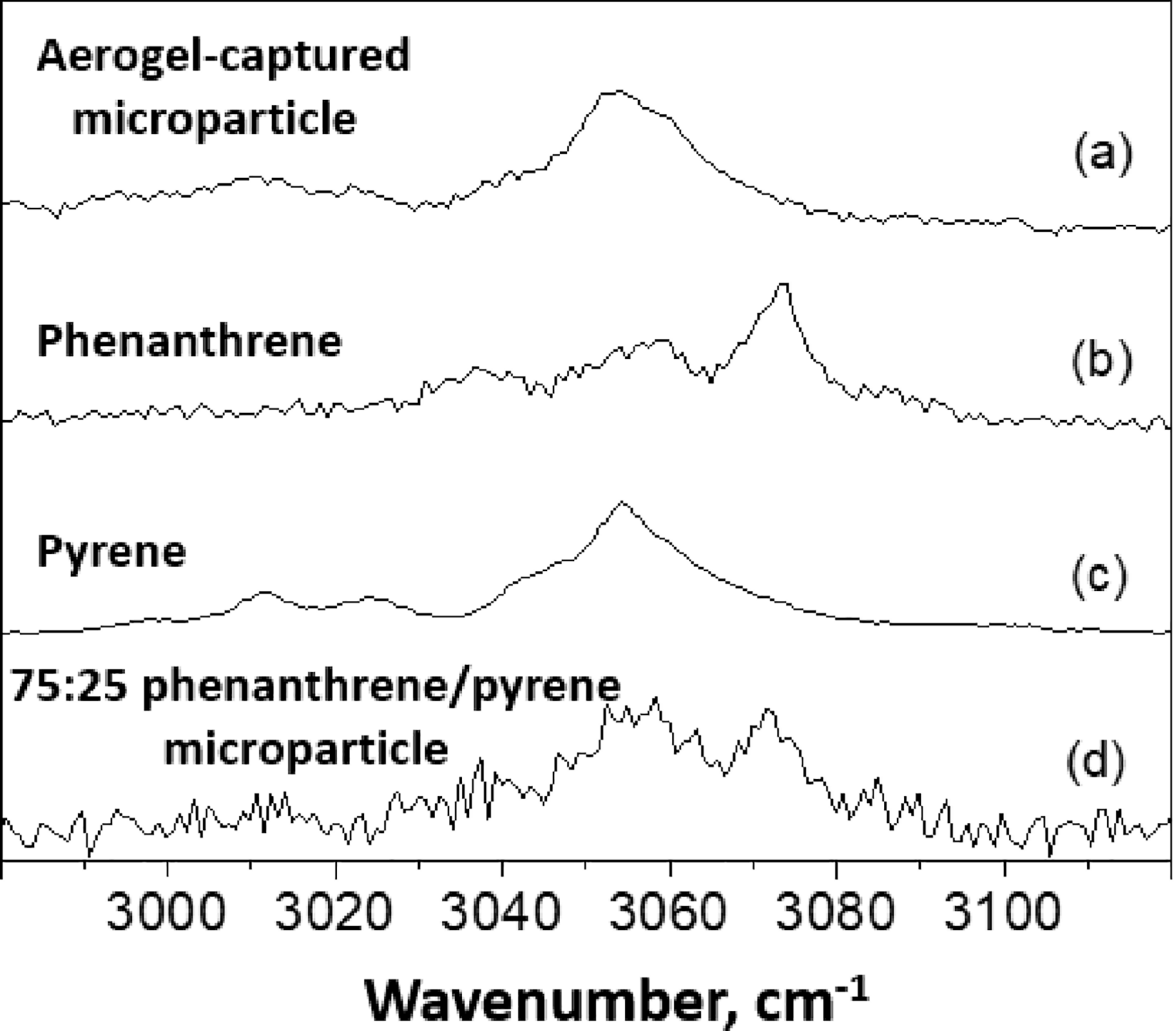
Background-subtracted partial Raman spectra recorded for (a) 75:25 phenanthrene/pyrene microparticles, (b) pyrene reference, (c) phenanthrene reference, and (d) an individual 75:25 phenanthrene/pyrene microparticle after its aerogel capture at approximately 1 km s^–1^. The original 75:25 phenanthrene/pyrene microparticles exhibit spectral features that are characteristic of both phenanthrene and pyrene, whereas the aerogel-captured microparticle only contains pyrene, with minimal evidence for the more volatile phenanthrene component.

Three regions of two individual aerogel-captured microparticles were examined, and multiple strong pyrene signals were observed for each region. Indeed, all the major bands observed in Figure 7 are also present in Figure 12, along with minor bands (also assigned to pyrene). However, there is no sign of any phenanthrene bands despite this being the major component of the original impinging microparticle. Moreover, the Raman spectrum recorded for the original microparticles using the same spectrometer contains phenanthrene bands of comparable intensity to that of the pyrene signals. Raman spectra were also recorded at around 3000 cm^–1^ (see Figure 13).

This high wavenumber region contains distinctive C–H stretching modes for both phenanthrene and pyrene. For phenanthrene, the strong band at 3072 cm^–1^ and weaker bands at 3034 and 3056 cm^–1^ are consistent with literature spectra.^35^ In contrast, the most prominent band in the corresponding pyrene spectrum is at 3054 cm^–1^, with weaker features at 3011 and 3024 cm^–1^. This is in good agreement with spectra reported by Cloustis et al.^47^ Again, the Raman spectrum recorded after firing 75:25 phenanthrene/pyrene hybrid microparticles into the aerogel target contains characteristic bands assigned to pyrene, but there is no evidence for phenanthrene, which constitutes the major component of the original projectile (see Figure 13). In summary, these Raman spectra suggest that phenanthrene does not survive aerogel capture at around 1 km s^–1^.

For such experiments, the peak shock pressure can again be calculated via the planar impact approximation, where the *C* and *S* coefficients depend on the aerogel density (ρ = 91.5 kg m^–3^). Applying Anderson’s formula^48^ yields *C* = 0.2825 m s^–1^ and *S* = 2.629. Determination of the individual peak pressures for phenanthrene and pyrene indicates the likely range for the actual peak pressure for the hybrid microparticles.

For aerogel capture at 0.99 km s^–1^, such a peak pressure ranges from 0.234 to 0.235 GPa. Given that the same microparticles exhibited only partial fragmentation when fired at an aluminum foil target at similar speed, the substantial change in the chemical composition observed for the aerogel-captured microparticles is most likely caused by differential thermal ablation of the more volatile phenanthrene component, which has a significantly lower boiling point compared to pyrene (332 °C vs 394 °C, respectively).

Recently, we fired pure phenanthrene microparticles at 2.07 km s^–1^ into an aerogel target.^20^ In this prior study, the aerogel density was 32 kg m^–3^, which resulted in an associated peak shock pressure of 0.212 GPa. Examining the microparticles captured within this lower density aerogel under UV irradiation also revealed fluorescence. However, in this case the corresponding Raman spectra merely comprised a strong fluorescent background with no characteristic phenanthrene bands. With the benefit of hindsight, this negative observation is consistent with the complete loss of the characteristic Raman bands for phenanthrene from aerogel-captured microparticles observed in the current study.

## Conclusions

We have exploited the relatively low melting point of phenanthrene to prepare two types of phenanthrene/pyrene microparticles by a highly convenient wholly aqueous melt processing route. A melting point phase diagram indicates the eutectic composition for such binary mixtures, which corresponds to 75 mol % phenanthrene. This particular composition has a eutectic temperature of 83 °C, which is well below the melting point of either phenanthrene or pyrene. Moreover, it is also below the boiling point of water, which enables spherical phenanthrene/pyrene microparticles to be readily prepared via hot emulsification in purely aqueous solution using either Morwet D-425 or poly(vinyl alcohol) as an emulsifier. For such formulations, the mean droplet size, and hence the final microparticle diameter, can be readily adjusted from 11 to 279 μm during high-shear homogenization simply by varying the stirring rate and the type of polymeric emulsifier. Hot-stage optical microscopy, ^1^H NMR spectroscopy and Raman microscopy studies confirm that the chemical composition of these phenanthrene/pyrene microparticles remains constant when prepared at their eutectic composition of 75 mol % phenanthrene.

The 75:25 phenanthrene/pyrene microparticles were fired into aluminum foil and aerogel targets in turn at around 1 km s^–1^ in each case. Under such conditions, sticking/fragmentation/cratering occurs when employing the former target,^39^ while microparticle penetration and capture are observed with the latter target.^30^ Understanding such behavior is critical when designing cosmic dust capture systems for future spacecraft. Importantly, Raman microscopy studies of microparticles captured within the aerogel target reveal complete thermal ablation of the major phenanthrene component, whereas the minor pyrene component remains intact. Knowledge of such a remarkable selection bias for simple PAHs is essential for any space mission that attempts to capture PAH-based grains intact within aerogel targets. For example, sampling dust particles within the icy plumes emanating from Enceladus could in principle involve either orbiting this moon at just a few hundred m s^–1^ or by undertaking a fly-by at an encounter velocity of 3–5 km s^–1^.^21^ The present study suggests that the successful collection of intact PAH-based dust particles is only likely to be achieved in the first scenario. It is also envisaged that further light gas gun experiments performed at higher hypervelocities (e.g., up to 7 km s^–1^) should enable identification of the maximum encounter velocity at which the most thermally stable PAH molecules can be captured intact.

In summary, these new PAH-based hybrid microparticles are expected to inform the calibration of the next generation of cosmic dust detectors for deployment in Low Earth Orbit, for the Gateway space station planned to orbit the Earth’s moon, and for missions to the moons of the outer planets.

## Supporting Information Available

Digital photograph of the hot-stage optical microscopy experimental setup; schematic representation of the two types of light gas gun experiments; digital images recorded for phenanthrene microparticles during hot-stage optical microscopy studies; ^1^H NMR spectra recorded for ten individual phenanthrene/pyrene microparticles (PDF)
