# Supplementary material for: Synthesis of Phenanthrene/Pyrene Hybrid Microparticles: Useful Synthetic Mimics for Polycyclic Aromatic Hydrocarbon-Based Cosmic Dust
Source: J Am Chem Soc. 2024 Jul 15;146(30):20802–13. doi: 10.1021/jacs.4c04330 (PMC11295189; doi:10.1021/jacs.4c04330)
Supplement: Supplementary file 1 — ja4c04330_si_001.pdf [file ja4c04330_si_001.pdf]

Supporting Information for:

Synthesis of phenanthrene/pyrene hybrid microparticles: useful synthetic mimics for polycyclic aromatic hydrocarbon-based cosmic dust

Emma E. Brotherton[a,$], Derek H. H. Chan[a,$], Steven P. Armes[a,*], Ronak Janani[b], Chris Sammon[b], Jessica L. Wills [c], Jon D. Tandy [d], Mark J. Burchell [c], Penelope J. Wozniakiewicz [c], Luke S. Alesbrook[c] and Makoto Tabata[e]

a. Dainton Building, Department of Chemistry, University of Sheffield, Brook Hill, Sheffield, South Yorkshire, S3 7HF, UK.

b. Materials and Engineering Research Institute, Sheffield-Hallam University, Sheffield, South Yorkshire, S1 1WB, UK.

c. School of Physics and Astronomy, University of Kent, Canterbury, Kent CT2 7NH, UK.

d. School of Chemistry and Forensic Science, University of Kent, Canterbury CT2 7NZ, UK.

e. Department of Physics, Chiba University, Chiba, Japan.

Summary of Contents

Figure S1. Digital photograph of the experimental set-up used for the hot-stage optical microscopy studies (see main manuscript).

Scheme S1. Schematic representation of the two types of light gas gun experiments undertaken in this study. Microparticles are loaded into the sabot, which is then fired and discarded in flight, so the microparticles alone strike the target. Microparticle fragmentation and crater formation occurs when using an aluminum foil target. In contrast, using an ultralow density aerogel target enables microparticles to be captured *in situ* (ideally with minimal thermal ablation).

Figure S2. Representative video stills recorded during hot-stage optical microscopy studies of an array of twenty pure phenanthrene microparticles (mean diameter = 202 µm) on heating from 100 °C to 102 °C.

Figure S3. Representative ^{1}H NMR spectra recorded for nine individual 3:1 phenanthrene/pyrene microparticles after their dissolution in d_6-acetone.

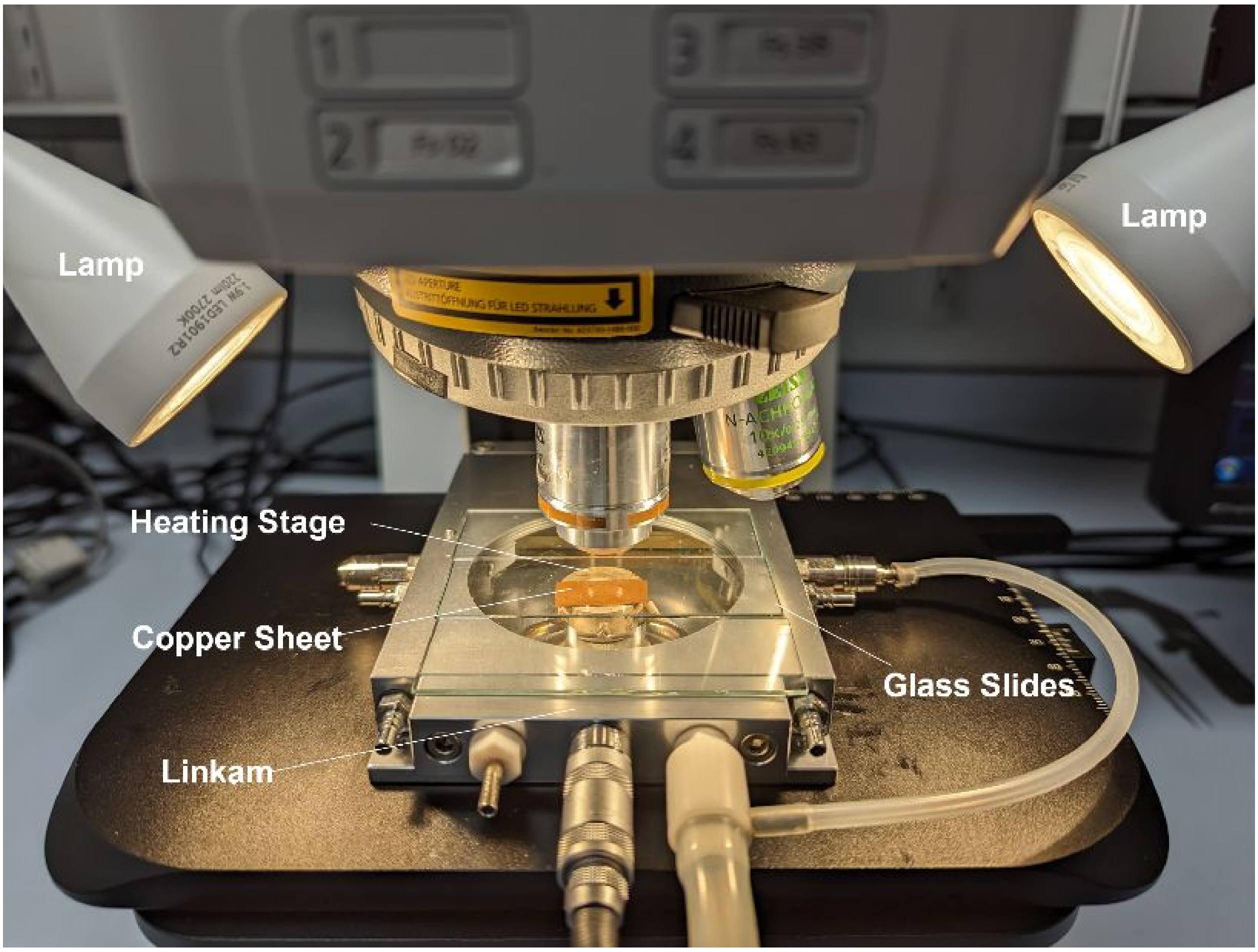

Figure S1. Digital photograph of the experimental set-up used for the hot-stage optical microscopy studies (see main manuscript).

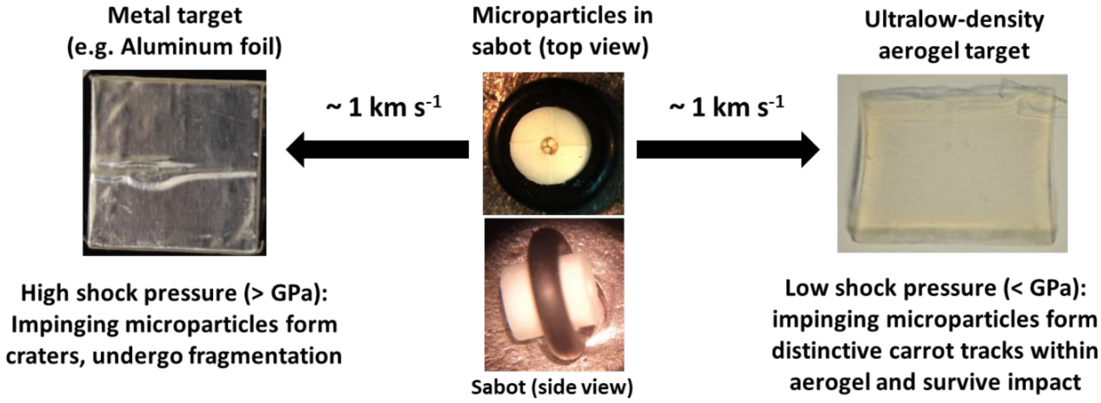

Scheme S1. Schematic representation of the two types of light gas gun experiments undertaken in this study. Microparticles are loaded into the sabot, which is then fired and discarded in flight, so the microparticles alone strike the target. Microparticle fragmentation and crater formation occurs when using an aluminum foil target. In contrast, using an ultralow density aerogel target enables microparticles to be captured *in situ* (ideally with minimal thermal ablation).

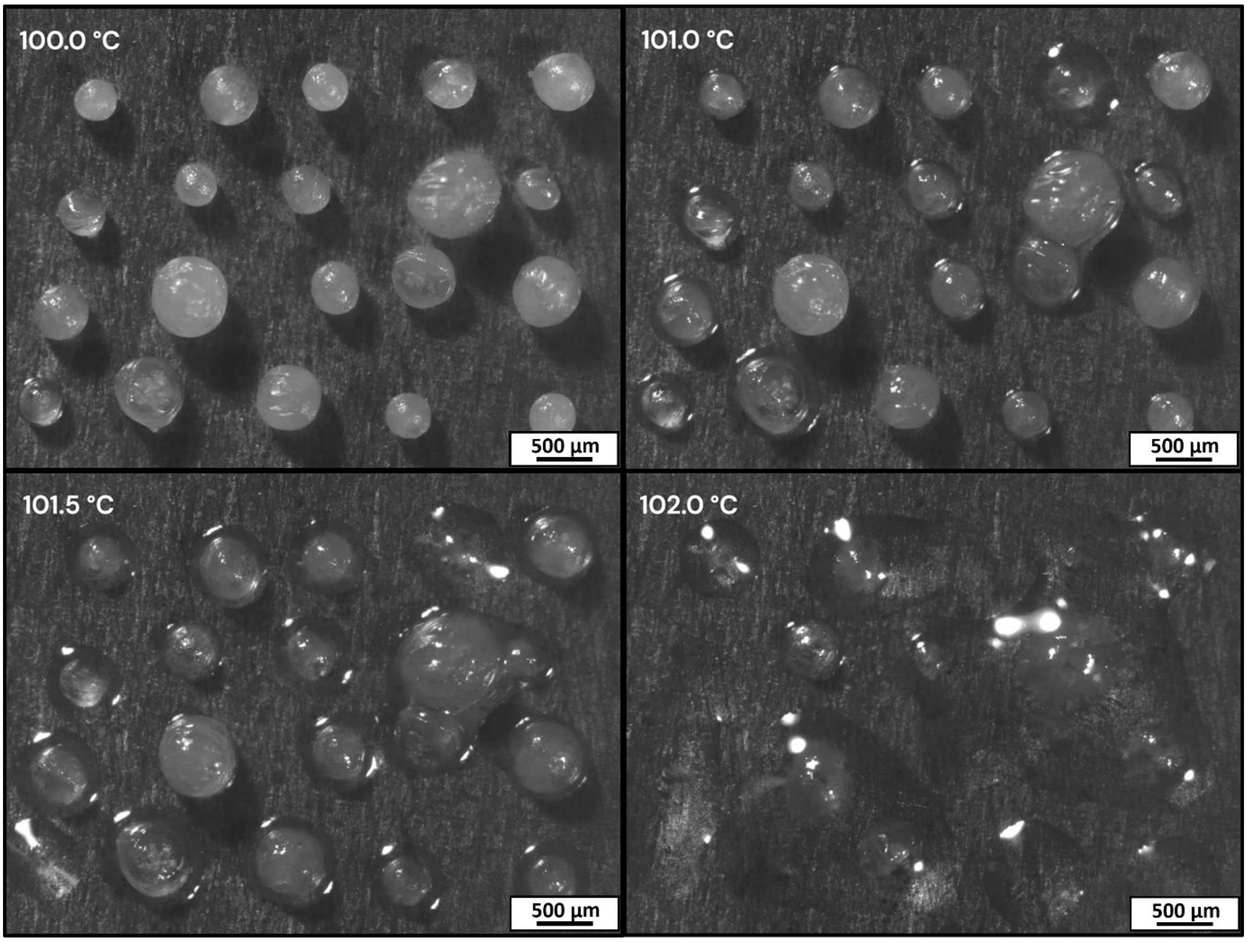

Figure S2. Representative video stills recorded during hot-stage optical microscopy studies of an array of twenty pure phenanthrene microparticles (mean diameter = 202 µm) on heating from 100 °C to 102 °C.

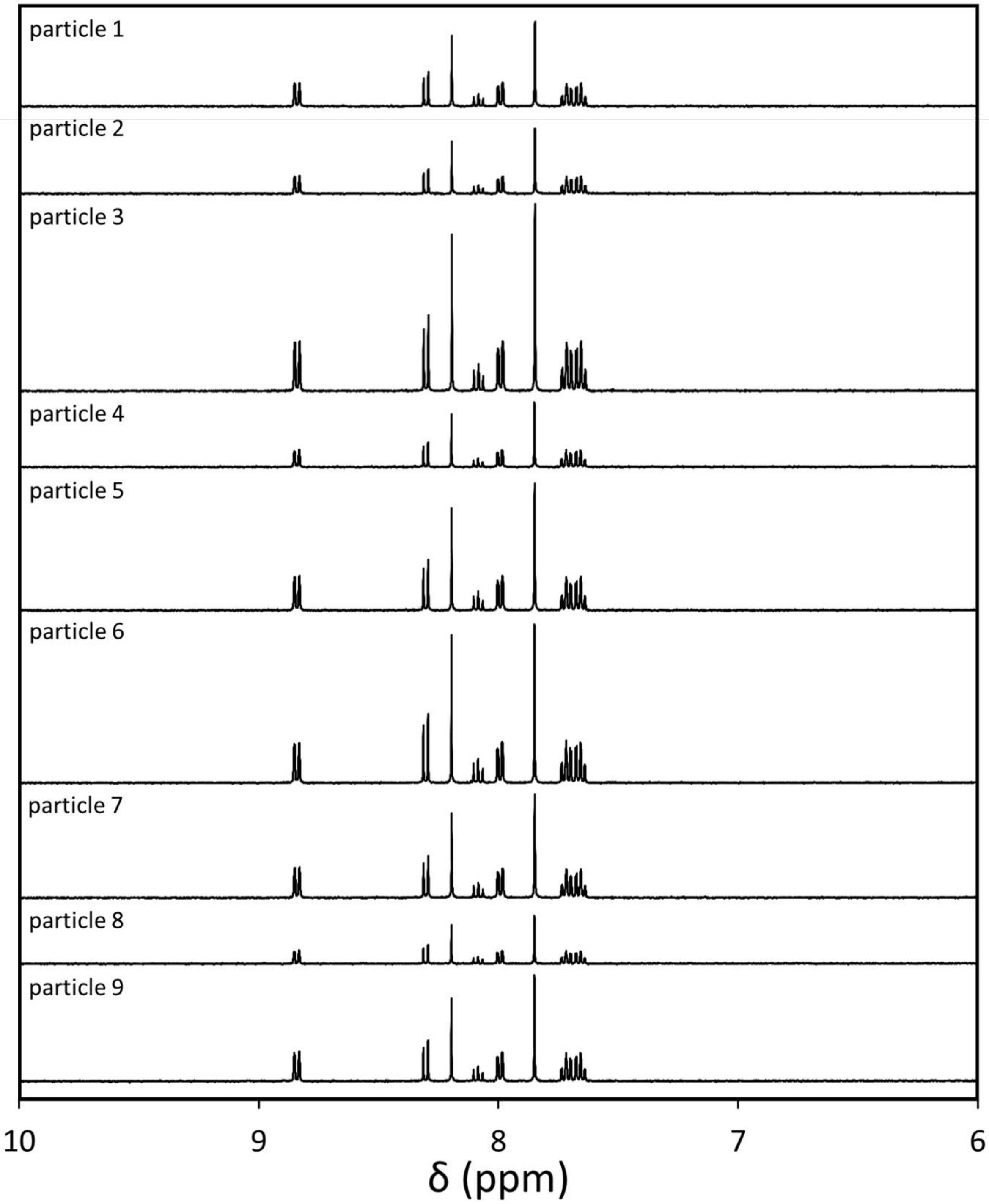

Figure S3. Representative ^{1}H NMR spectra recorded for nine individual 3:1 phenanthrene/pyrene microparticles after their dissolution in d_6-acetone. Analysis of such spectra indicate that these nine microparticles have essentially the same chemical composition within experimental error.
